# Characteristics of Traffic Flow at Nonsignalized T-Shaped Intersection with U-Turn Movements

**DOI:** 10.1155/2013/856416

**Published:** 2013-04-04

**Authors:** Hong-Qiang Fan, Bin Jia, Xin-Gang Li, Jun-Fang Tian, Xue-Dong Yan

**Affiliations:** MOE Key Laboratory for Urban Transportation Complex Systems Theory and Technology, Beijing Jiaotong University, Beijing 100044, China

## Abstract

Most nonsignalized T-shaped intersections permit U-turn movements, which make the traffic conditions of intersection complex. In this paper, a new cellular automaton (CA) model is proposed to characterize the traffic flow at the intersection of this type. In present CA model, new rules are designed to avoid the conflicts among different directional vehicles and eliminate the gridlock. Two kinds of performance measures (i.e., flux and average control delay) for intersection are compared. The impacts of U-turn movements are analyzed under different initial conditions. Simulation results demonstrate that (i) the average control delay is more practical than flux in measuring the performance of intersection, (ii) U-turn movements increase the range and degree of high congestion, and (iii) U-turn movements on the different direction of main road have asymmetrical influences on the traffic conditions of intersection.

## 1. Introduction

**Figure 1:**
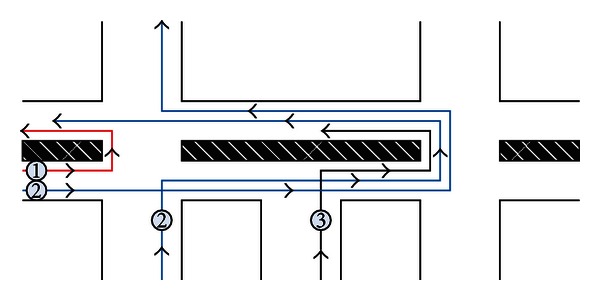
Origination of U-turn movement at the intersection. Three types vehicles need make U-turn movements at the nearest intersections: (1) vehicles which move the opposite direction; (2) vehicles which are forbidden to make direct left-turn movement at the intersection; (3) vehicles which turn left from a minor driveway.

U-turn movements increase the complexity of urban intersections. However, it is applied more and more widely. The main reasons are forbidding left-turn movements at the intersection and no median opening on the road.  Figure 1 displays the process of U-turn movements.

Recently, The effects of U-turn movements on the safety and operation of intersections have attracted great research interests. These studies can be substantially categorized into using the analytical models [1–8] and simulation techniques [9–23].

Some researchers investigated the capacity of signalized intersections permitting U-turn movements [1–4]. The related conclusions were used in Highway Capacity Manual (HCM 2010). Several studies [5, 6] have analyzed the characteristics of U-turn movements at the unsignalized intersections from four aspects (i.e., headway acceptance, impedance effects of minor movements, conflicting traffic volume, and shared-lane capacity of the major street exclusive left-turn lane). Liu et al. [7] evaluated the impacts of indirect driveway left-turn treatments on traffic operations at signalized intersections. Guo et al. [8] have developed a negative-binomial model to predict U-turn volume on a left-turn approach at a signalized intersection during weekday peak periods.

Analytical models adapt to analyze a single intersection. However, it is not suitable for more complex cases, for example, two or more intersections. Traffic simulation techniques can compensate the disability of analytical models. One kind of simulation approaches are based on some commercial softwares, including CORSIM, AIMSUN, and VISSIM [9–12]. CA models were also used to investigate the characteristics of traffic flow at intersections of different types [13–23]. The intersection types include roundabout, cross intersection, and T-shaped intersection. At a T-shaped intersection, Li et al. [19] investigated the influence of the left-turning car on the whole traffic situation by introducing the priority probability of the through vehicle; Wu et al. [20] analyzed the interactions between vehicles on different lanes and effects of traffic flow states of different roads on capacity of nonsignalized system; Li et al. [21] considered three input flows and two left turnings to study the traffic behaviors under two crash avoiding rules; Ding et al. [22] investigated and compared the phase diagram, the capacity and the average travel time of two different signal controlling systems. Fan et al. [23] explored the characteristic of traffic flow at the nonsignalized T-shaped intersection with all directional vehicles. 

However, these studies did not measure the performance of intersections by the average control delay but by the flux for convenience. This is not practical. In addition, the effect of U-turn movements at nonsignalized intersections was also ignored. For these reasons and based on the former work in [23], this paper proposes a new CA model to characterize the nonsignalized T-shaped intersection with U-turns. For this, new avoiding conflicts and gridlock avoiding rules are developed, and the average control delay (while not flux) is introduced as the performance index. The remainder of this paper is organized as follows. In Section 2, the model is illustrated in detail. In Section 3, the numerical and analytical results are analyzed. Conclusions are summarized in Section 4.

## 2. Model

**Figure 2:**
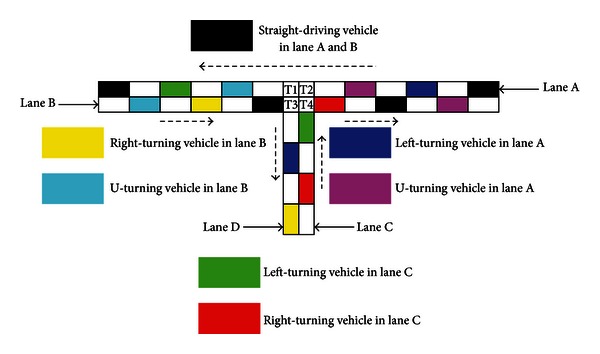
Type of nonsignalized T-shaped intersection.

The geometric design of analyzed T-shaped intersection is illustrated in Figure 2. That is the same as in the literature [19–21]. Each of the approaches has one lane. The major street is consisted of lane A and lane B. The minor street is composed by lane C and lane D. Vehicles move in the right lane. The length of major street is *L*, and minor street is *L*/2 − 1. The intersection is made up of four cells, T1, T2, T3, and T4. There are seven types of vehicles: (a) straight-driving vehicles on lane A and B, *s* = 1; (b) left-turning vehicles on lane A, *s* = 2; (c) U-turning vehicles on lane A, *s* = 3; (d) right-turning vehicles on lane B, *s* = 4; (e) U-turning vehicles on lane B, *s* = 5; (f) left-turning vehicles on lane C, *s* = 6; (g) right-turning vehicles on lane C, *s* = 7. Here, *s* denotes the type of vehicles.

### 2.1. The Rules of Vehicle Moving on the Road

NaSch model [24] is used to simulate the vehicle movement. Although simply, it can reproduce many basic phenomena in realistic traffic, such as the start-stop waves. In NaSch model, time and space are discrete. The road is divided into *L* cells. Each cell has two conditions, occupied by one vehicle or empty. The vehicle speed can be 0, 1, 2,…, *v*_max⁡_; here *v*_max⁡_ is the maximum speed. The update rules of NaSch model are as follows:

 step 1: acceleration, *v*_*n*_ → min⁡(*v*_*n*_ + 1, *v*_max⁡_);
 step 2: deceleration, *v*_*n*_ → min⁡(*v*_*n*_, *d*_*n*_);
 step 3: randomization, *v*_*n*_ → max⁡(*v*_*n*_ − 1,0) with probability *p*;
 step 4: position update, *x*_*n*_ → *x*_*n*_ + *v*_*n*_.

Here, *v*_*n*_ and *x*_*n*_ denote the speed and position of vehicle *n*, respectively; *d*_*n*_ = *x*_*n*−1_ − *x*_*n*_ − *l* is the number of empty cells in front of vehicle *n* − 1, and *l* is the length of vehicle *n*; *p* is the randomization probability.

### 2.2. The Rules of Avoiding Conflict at the Intersection

Due to the intersection cell that can be occupied only by one vehicle, many potential conflicts may occur when vehicles approach to the intersection. Four types of conflicts are classified.

- At T1, the straight-driving or left-turning vehicles on lane A or the left-turning vehicles on lane C may conflict with the U-turning vehicles on lane B.
- At T2, the vehicles on lane A may conflict with left-turning on lane C.
- At T3, the vehicles on lane B may conflict with left-turning on lane A.
- At T4, the U-turning vehicles on lane A, the straight-driving vehicles on lane B, and the vehicles on lane C may conflict with each other.

In order to prevent accidents, some control rules should be applied when the potential conflicts occur. The velocity, position, and type of the first vehicle upstream cell T1 (include T1) on lane A are denoted as *x*_A_, *v*_A_, and *s*_A_, respectively; those of the first vehicle upstream cell T4 on lane B are *x*_B_, *v*_B_, and *s*_B_, and those of the first vehicle on lane C (T4 as the *L*/2 cell on lane C) are *x*_C_, *v*_C_, and *s*_C_. When the potential conflict occurs, the time, which the first vehicle that upstreams the intersection on each lane needs to reach the conflict cell, is calculated. The times are denoted as *t*_A_, *t*_B_, and *t*_C_, respectively. The conflict cell will be occupied by the vehicle which uses less time to reach the conflict cell. If the times are equal, the priority vehicle will occupy the conflict cell. According to the Highway Capacity Manual (HCM 2000), the priority of right of way given to each traffic stream can be identified as follows. Movements of rank 1 include through traffic stream on the major street and right-turning traffic stream from the major street. Movements of rank 2 include left-turning traffic stream from the major street and right-turning traffic stream onto the major street. Movements of rank 3 include left-turning traffic stream from the minor street. Movements of rank 4 include U-turning traffic stream from the major street.

Three types of gridlock in the system are identified (a) the cell T1 is occupied by a left-turning vehicle on lane A and the cell T3 is occupied by a U-turning vehicle on lane B at the same time; (b) the cell T2 is occupied by a U-turning vehicle on lane A and the cell T4 is occupied by a left-turning vehicle on lane C at the same time; (c) the cell T1 or the cell T2 is occupied by a left-turning vehicle on lane A, the cell T3 is occupied by a straight-driving vehicle on lane B, and the cell T4 is taken up by a left-turning vehicle at the same time. In order to avoid the gridlock, the following rules corresponding to above types are used, respectively: (a) if the cell T1 or T2 has been or will be occupied in the next time step by a left-turning vehicle on lane A, the U-turning vehicle on lane B is not allowed to enter into the cell T3; if the cell T3 has been occupied by the U-turning vehicle on lane B, the left-turning vehicle on lane A is forbidden to enter into the cell T1; (b) if the cell T4 has been or will be occupied in the next time step by a left-turning vehicle on lane C, the U-turning vehicle on lane A is not allowed to enter into the cell T2; if the cell T2 has been occupied by the U-turning vehicle on lane A, the left-turning vehicle on lane C is forbidden to enter into the cell T4; (c) if the cell T1 or T2 has been or will be occupied in the next time step by a left-turning vehicle on lane A, the left-turning vehicle on lane C is not allowed to enter into the cell T4; if the cell T4 has been occupied by the left-turning vehicle on lane C, the left-turning vehicle on lane A is forbidden to enter into the cell T1 and T2.

The system operates as follows. Firstly, the velocities of all vehicles are updated. Then, the conflicts and gridlock are identified and disposed. At last, the positions of all vehicles are updated. The simulations are carried out under open boundary condition. At each time step, we check the position of the last vehicle on each lane, which is represented as *x*_*λ*_^last^. If *x*_*λ*_^last^ > *v*_max⁡_ + *l*, a new vehicle with the maximum velocity *v*_max⁡_ is injected with inflow rate *p*_*λ*_ at the position min⁡(*v*_max⁡_, *x*_*λ*_^last^ − *v*_max⁡_). Here, *λ* = A, B, C. The vehicle on lane A is set as a left-turning vehicle with probability *p*_LA_ and a U-turning vehicle with probability *p*_UA_. The vehicle on lane B is set as a right-turning vehicle with probability *p*_RB_ and a U-turning vehicle with probability *p*_UB_. The vehicle on lane C is set as a left-turning vehicle with probability *p*_LC_. If the position of first vehicle on lane A, B, and D is larger than the length of lane, the vehicle will be removed, and the following vehicle will be the new leading vehicle.

## 3. Simulation and Discussion

In the simulation, *L* = 1000, *l* = 2, *p* = 0.3, *p*_LA_ = 0.1, *p*_RB_ = 0.1, *p*_C_ = 0.1, *p*_LC_ = 0.5, and *v*_max⁡_ = 6. Each cell corresponds to 3.75 m; thus, the length of a vehicle is 7.5 m. The first 50000 time steps are discarded to avoid the transient behaviors. The detector is set at the 490th cell on each lane upstream the intersection to obtain the flux by counting the number of vehicles in 20000 time steps. The flux of lane A, B, and C are denoted as flux_A_, flux_B_, and flux_C_ which represent the average flux of each time step. The total flux of T-shaped intersection is the sum of flux_A_, flux_B_, and flux_C_, which is denoted as flux_All_. The travel time detectors are placed relatively far upstream and downstream of the intersection to better capture the delays of each movement at the intersection. Delay data are extracted for the 20000 time step. The averageDelay_A_, averageDelay_B_, averageDelay_C_ and averageDelay_All_ denote the average control delay of each vehicle on lane A, B, C and the intersection.

### 3.1. Performance of Flux and Average Control Delay

**Figure 3:**
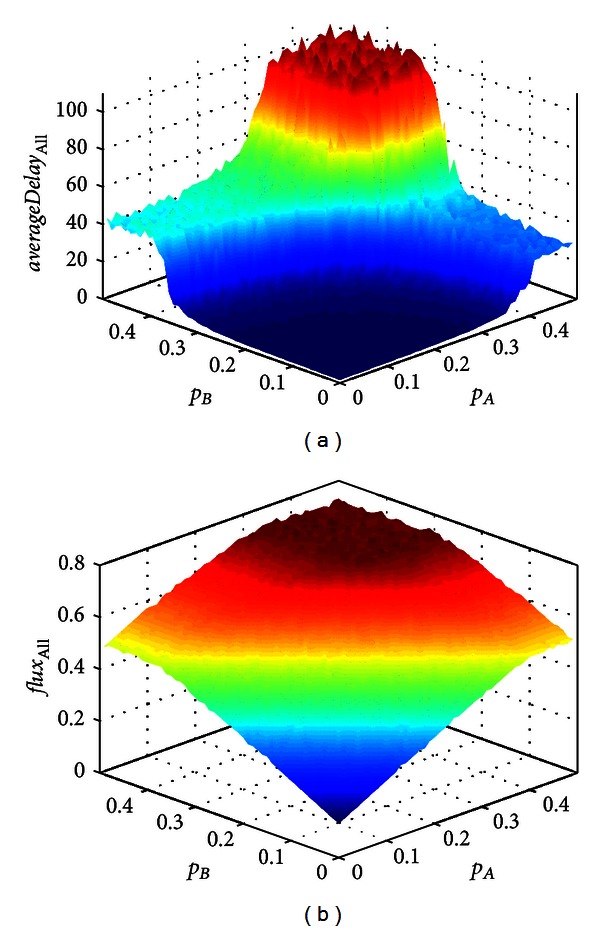
Performance of (a) average control delay and (b) flux of intersection in space (*p*_A_, *p*_B_). Assumed the parameter is *p*_UB_ = 0.

**Figure 4:**
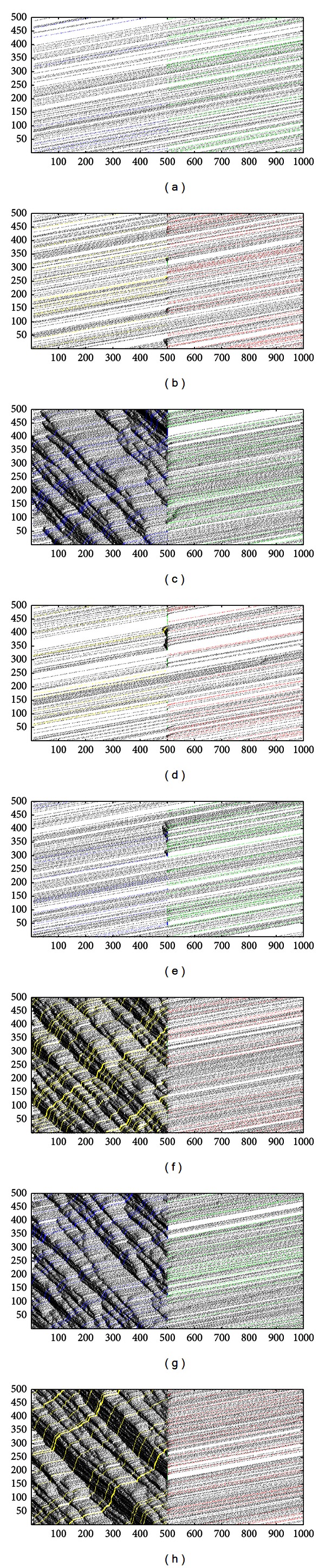
Spatiotemporal diagram of lane A (a), (c), (e), and (f) and lane B (b), (d), (f), and (h) with different inflow rates.

The flux and average control delay can reflect the traffic condition of intersection. But their performance is different.  Figure 3 shows (a) the flux and (b) the average control delay of intersection in space (*p*_A_, *p*_B_). The simulation system possesses a certain ability of self-organization. So, it can suffer a limited fluctuate of inflow rates and remain a small change.  Figure 3(a) contains four plane regions and three transitional regions which are very narrow and steep. The plane region means the average control delay keeps a stable value with the vary of inflow rates. The transitional region means the average control delay increases dramatically with the increase of inflow rates. If the inflow rates exceed the critical value, the average control delay will increase dramatically and then reach a new stable value. It means the simulate system can exchange from one equilibrium state to another equilibrium state quickly. When the inflow rates are very high, the simulate system can remain the equilibrium state, but it appears a little obvious fluctuation. A curved surface is seen in Figure 3(b). According to the change of color, it can be known that the flux is increasing steadily with the increase of inflow rates and then becomes to be constant value. It means the intersection capacity has a maximum value under a certain condition. It could not be changed by increasing the inflow rates and reflected the flexible equilibrium states. Compared the Figures 3(a) and 3(b), both of them can reflect the performance of intersection. The flux can reflect the intersection state in macrolevel such as free flow and jam flow. But the degree of traffic jam could not be known. The average control delay can reflect the condition of a vehicle movement at the intersection in microlevel. According to the average control delay, the free flow and degree of traffic jam can be classified. One plane region represents one particular state of intersection. And the average control delay can tell the driver how long he will waste to pass the intersection. It is very useful for a driver decide whether pass the intersection. The traffic management department can decide whether implements some traffic measures according to the average control delay. So, the average control delay can reflect the performance of intersection more reasonable than flux.  Figure 4 shows the spatiotemporal diagram of lane A (a), (c), (e), and (f) and lane B (b), (d), (f), and (h) with different inflow rates. Each subgraph represents the traffic condition of each plane region in Figure 3(a). The free flow or congestion flow of each lane can be observed in Figure 4.

### 3.2. Influence of U-Turn Movements

The influence of U-turn movements on the intersection is investigated in this part. The average control delay is used to reflect the performance of intersection.

**Figure 5:**
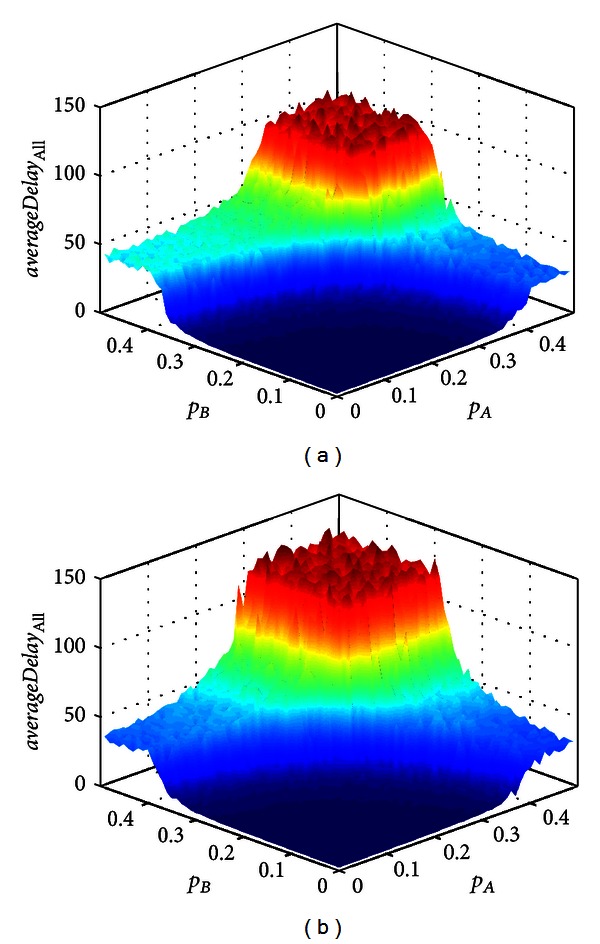
Influence of U-turns on the performance of intersection in space (*p*_A_, *p*_B_). In the cases of (a) *p*_UA_ = *p*_UB_ = 0 and (b) *p*_UA_ = *p*_UB_ = 0.05.

Figure 5 shows the influence of U-turn movements on the performance of intersection under different inflow rates in the cases of (a) *p*_UA_ = *p*_UB_ = 0, (b) *p*_UA_ = *p*_UB_ = 0.05. Compared Figures 5(a) and 5(b), the traffic conditions become very sensitive to U-turn movements with the increase of inflow rates. A little increase of U-turn movements causes a large increase of average control delay. So, this plane region range, when the inflow rates are large enough, enlarged due to the appearance of U-turn movements. That is because the appearance of U-turn movements makes the traffic condition of intersection more worse, and more potential conflicts with other traffic streams are occurred. The vehicles must stop more frequently to avoid collisions. So, they spend more time to pass the intersection. That means it caused more control delay. It is not obvious when the inflow rates are small. But it is very obvious when the inflow rates are large. Therefore, reducing the U-turn movements can improve the traffic condition when the inflow rates are large.

**Figure 6:**
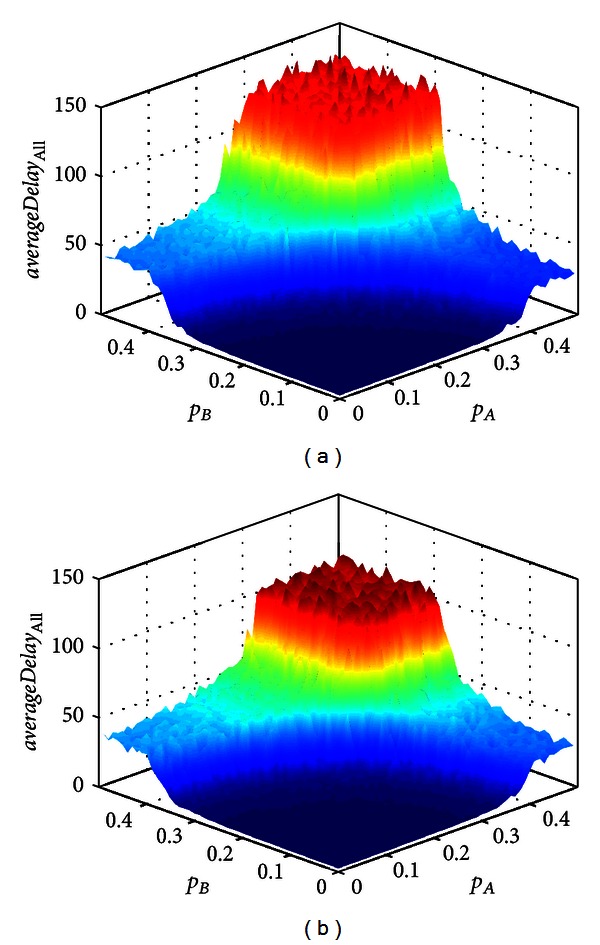
Influence of U-turns at different lane on the performance of intersection in space (*p*_A_, *p*_B_). In the cases of (a) *p*_UA_ = 0.05, *p*_UB_ = 0, (b) *p*_UA_ = 0 and *p*_UB_ = 0.05.

Although the lane A and lane B are the major streets, the influence of U-turn movements on the intersection is different.  Figure 6 shows the influence of U-turn movements at lane A and lane B on the performance of intersection in the cases of (a) *p*_UA_ = 0.05, *p*_UB_ = 0, (b) *p*_UA_ = 0, *p*_UB_ = 0.05. Compared Figures 6(a) and 6(b), the traffic conditions become more sensitive to U-turn movements at lane A than lane B with the increase of inflow rates. When the inflow rates are large enough, the average control delay caused by U-turn movements at lane A is larger than that at lane B. This plane region range with U-turn movements at lane A is also larger than at lane B. The reason is that the U-turning vehicles at lane A pass the intersection may conflict with vehicles on lane B and C. But the the U-turning vehicles at lane B just may conflict with vehicles on lane A. So, the U-turns at lane A make more conflict with other traffic streams than lane B. It suggests that control the U-turn movements at lane A can improve the traffic condition when the inflow rates are large.

## 4. Conclusion

In this paper, a new CA model is proposed to characterize the nonsignalized T-shaped intersection with U-turn movements. For this, new avoiding conflicts and gridlock avoiding rules are defined, and the average control delay (while not flux) is introduced as the performance measure. Simulations based on the present new CA model are executed. Three findings can be concluded from the simulation results: firstly, compare with flux, the average control delay is more practical to measure the performance of intersection; secondly, when the inflow rates are large, U-turn movements can worsen the traffic condition of intersection, that is, increasing both range and degree of high congestion; finally, U-turn movements on the different direction of main-road have asymmetrical influences on the traffic condition of intersection, for example, in the present example, the U-turn movements on lane A has more influence than that on lane B. Consequently, to improve the traffic condition of intersection, U-turn movements should be restricted when the inflow rates are large enough.
